# Supplementary material for: DNA Methylation Is Correlated with Gene Expression during Diapause Termination of Early Embryonic Development in the Silkworm (Bombyx mori)
Source: Int J Mol Sci. 2020 Jan 20;21(2):671. doi: 10.3390/ijms21020671 (PMC7013401; doi:10.3390/ijms21020671)
Supplement: Supplementary file 1 [file ijms-21-00671-s001.zip › Supplementary-IJMS/Supplementary Table S1.docx]

**Table S1. Primers used in experiments**

| Gene ID | Forward primer (5’-3’) | Reward primer (5’-3’) | Product size |
| --- | --- | --- | --- |
| RT-qPCR _101742044 | TATTGCCCTGTGGCGTTGA | GCGGTGTCTGGTGGTCTTCTA | 271 |
| RT-qPCR _101739131 | TTGGCTTATGGCGACGATG | GCAAACTCCTTTCCGCTCAG | 260 |
| RT-qPCR _101739208 | ACAAGCAGGAGAAGTCATCGG | ACGGGCATGGTGACATAGAGT | 256 |
| RT-qPCR _110385781 | TGGCATCGCAAGAAGCAG | CATACGGCGCACTAAAGCAC | 129 |
| RT-qPCR _101740063 | TTCGATGAATATCCGACACCTT | GCTGGCACGACACCACTTT | 150 |
| RT-qPCR _101741941 | TTCCACTGAAGGGAGACTGCT | GGGCTTATCTTAGACGATTACCAA | 179 |
| McrBC-qPCR_101739208 | GGCTGTTCGATACCGTTCTG | TTTCGTCACTTAGCGTCTCCTT | 447 |
| McrBC-qPCR_110385781 | ACTGCCCCAAGAAGTTCAAAG | TTACCAGGCCAAGACAGACAAG | 434 |
| BS-PCR_ 101739208 | AAGATGTTTATAGGTAAAGGTT  TTG | CTAATACCCATAAAAAATCCTCAT  TTA | 461 |
